# The Mental Health Implications of Obstetric Brachial Plexus Injuries (OBPI) on Parents

**DOI:** 10.34763/jmotherandchild.20232701.d-23-00024

**Published:** 2023-11-22

**Authors:** Catherine Miller, Karan Dua, Nathan N. O’Hara, Catherine C. May, Joshua M. Abzug

**Affiliations:** Department of Orthopaedics, University of Maryland School of Medicine, Baltimore, MD, USA; Department of Orthopaedic Surgery & Rehabilitation Medicine, SUNY Downstate Medical Center, Brooklyn, NY, USA

**Keywords:** OBPI, parents, depression, PTSD, NICU

## Abstract

**Background:**

Obstetric brachial plexus injuries (OBPI) can have mental health implications on parents coping with this injury to their newborn. The purpose of this study was to assess the mental health of mothers with newborns with an OBPI and identify resources that can help screen and treat mental health needs.

**Material and methods:**

Three groups of mothers were prospectively given a self-reported survey: 1) Newborns with OBPI; 2) Newborns in the nursery without OBPI; 3) Newborns in the neonatal intensive care unit (NICU). The survey consisted of demographic questions, the PHQ-9 and PCL-S screening tools, and parents’ exposure to community violence, family support and use of drugs or alcohol.

**Results:**

Fifty-seven mothers were prospectively enrolled, and 30% (17/57) of mothers screened in for post-traumatic stress disorder (PTSD). OBPI mothers had significantly higher rates of PTSD symptoms when compared to mothers of children in the full-term nursery (difference = 36.4%; p < 0.01). No statistically significant difference was found between groups regarding depression symptoms.

**Conclusions:**

OBPI can be very difficult to cope with for parents and family members. Forty-two percent of mothers with newborns with OBPI or children in the NICU screened in for PTSD symptoms. OBPI clinics should be staffed similarly to the NICU with clinical social workers to appropriately screen and treat parents with PTSD and depression symptoms.

## Background

Obstetric brachial plexus injuries (OBPI) can be a traumatic experience, both physically and mentally, leading to substantial mental health implications for families. OBPI are commonly associated with a shoulder dystocia delivery, in which the neonate's arm cannot pass through the vaginal canal, potentially leading to a traumatic delivery. The current incidence of OBPI is reported at 1.0–3.0 per 1000 live births [1,2].

The psychological effects of childbirth have historically been overlooked in public discourse and scientific research, but only in more recent years have researchers and clinicians begun to acknowledge childbirth as a traumatic event [3]. Childbirth can pose an imminent threat of severe injury or death to both a mother and newborn, which can lead to the development of mental disorders such as post-traumatic stress disorder (PTSD) [4]. The overall maternal birth-related PTSD rate from a recent meta-analysis conducted by Heyne and colleagues was found to be 4.7%. This rate was found to be higher in targeted groups at 6.8%, which includes those with maternal birth complications and traumatic birth events [5].

Traumatic deliveries, such as can be seen with a shoulder dystocia, may be associated with unrecognised and untreated psychological conditions involving not only the mother, but also her partner and other family members [6,7]. Parents may have unresolved feelings about the delivery and their newborn's injury including feelings of sadness, anger, anxiety, and decreased quality of life [1, 8,9,10,11,12,13,14]. However, the incidence of depression and PTSD in parents with an infant born with a brachial plexus injury has not been studied to date.

The purpose of this study was to assess the risk of depression and PTSD among mothers of newborns with OBPI compared to mothers with infants hospitalised in the neonatal intensive care unit (NICU) or newborn nursery.

## Material and Methods

Following Institutional Review Board approval, a prospective case-control study was performed to assess the mental health implications of having a child born with an OBPI or requiring a NICU hospital stay versus having a newborn baby without an OBPI in the newborn nursery. Three groups of mothers were given a 10-minute self-reported survey: 1) newborns with OBPI; 2) newborns in the newborn nursery without an OBPI; 3) newborns in the NICU. Mothers of a newborn with an OBPI were surveyed in outpatient OBPI clinics, while mothers of healthy newborns or those requiring a NICU stay were surveyed while the child was still in the hospital. Mothers were excluded if they did not speak English as their primary language or if they were not the biological mother of the newborn.

The survey consisted of demographic questions followed by the PHQ-9 and PCL-S screening tools. The PHQ-9 is a 10-item depression survey that asks respondents to rate their mood symptoms on a Likert scale, and the PCL-S is a 17-item PTSD survey that rates symptoms on a Likert scale. The questions were based on the DSM-V PTSD diagnostic criteria. Additionally, participants were queried about their education, marital status, employment, if they had any exposure to community violence or drug use and whether or not they felt supported by family and friends.

### Statistical Analysis

Continuous variable characteristics of the participants were described using means and standard deviations and medians and interquartile ranges depending on the distribution of the data. Categorical variables were described using counts and proportions. Associations between the participants’ characteristics and their child's post-natal state (OBPI vs. NICU vs. full-term nursery) were calculated using ANOVA for continuous variables and Fisher's Exact Test for categorical variables. The comparison among depression and PTSD events and the child's post-natal status were analysed using Fisher's Exact test. The significance level was set to *p* < 0.05. All statistical analyses were conducted using JMP Version 12 (SAS Institute, Cary, NC).

## Results

Fifty-seven mothers participated in the survey, including 24 mothers of a newborn with an OBPI, 14 mothers of a newborn in the NICU, and 19 mothers of a child in the newborn nursery. (Table 1). No statistically significant differences were found among respondents in ethnicity, available family support, alcohol or drug use, education level and employment status (*p* > 0.05).

**Table 1.** Patient Demographics.

**Variable**	**OBPI (n=24)**	**NICU (n=14)**	**FTN (n=19)**	***P*-Value**
Age of child at time of screening, Median (IQR)	27 (1–45)	1 (0.1.25)	0 (0–0)	0.0017^*^
Ethnicity, n (%)				0.5103
African-American	17 (70.8)	10 (71.4)	12 (63.2)	
White	4 (16.7)	4 (28.6)	6 (31.6)	
Other	3 (12.5)	-	1 (5.3)	
Recently witnessed violence [None], n(%)	12 (100.0)	11 (84.6)	18 (100.0)	0.0889
Support available [Yes], n(%)	7 (58.3)	8 (61.5)	13 (72.2)	0.7169
Drug use [None], n(%)	11 (91.7)	12 (92.3)	9 (50.0)	0.4686
Married [Yes], n(%)	5 (41.7)	5 (38.4)	2 (28.6)	0.5882
Education Level, n(%)				0.8141
Less than High School	-	-	1 (5.6)	
High School	4 (33.3)	4 (30.8)	7 (38.9)	
Some College	4 (33.3)	3 (23.1)	5 (27.8)	
College Degree	4 (33.3)	4 (30.8)	3 (16.7)	
Graduate School	-	2 (15.4)	2 (11.1)	
Employed, n(%)				0.3499
Full-time	6 (50.0)	4 (30.8)	11 (61.1)	
Part-time	1 (8.3)	2 (15.4)	-	
Not working	5 (41.7)	7 (53.8)	7 (38.9)	
PCL score, mean (SD)	31.0 (13.1)	31.8 (17.7)	21.0 (5.05)	0.0191^*^
PHQ-9 score, mean (SD)	7.5 (7.6)	8.5 (7.5)	2.2 (3.0)	0.0105^*^

SD = Standard Deviation; OBPI = obstetric brachial plexus injury; NICU = neonatal intensive care unit; FTN = full-term nursery; PCL = Post-Traumatic Stress Disorder Checklist; PHQ-9 = Patient Health Questionnaire.

Thirty percent (17/57) of mothers screened in for PTSD related symptoms, 43% (6/14) were NICU mothers, 42% (10/24) were OBPI mothers, and 5% (1/19) were newborn nursery mothers (Tables 2, 3). No statistically significant differences were found in PTSD screening rates of OBPI mothers compared to NICU mothers (difference = 1.2%; *p* = 1.00). However, OBPI mothers had significantly higher rates of PTSD related symptoms when compared to mothers of children in the full-term nursery (difference = 36.4%; *p* < 0.01). Forty-six mothers completed the PHQ-9 section of the survey for depression; 9% (4/46) of mothers screened in for depression related symptoms, 15% (2/13) were NICU mothers and 14% (2/14) were OBPI mothers. Similar to PTSD comparisons, there was no statistically significant difference observed in rates of depression symptoms between NICU and OBPI mothers (difference = −1.1%; *p* = 1.00) (Tables 4, 5). OBPI mothers trended towards having a higher rate of depression related symptoms than full-term nursery mothers (2 vs. 0) (difference = 14.3%; *p* = 0.17).

**Table 2.** Comparison of PTSD rates in mothers after obstetric brachial plexus injury to mothers of children in a neonatal intensive care unit and mothers of children in a full-term nursery.

	**N**	**PTSD Event Rate**	**Difference (95% CI)**	***P*-Value**
OBPI	24	41.7 %	−1.2% (−32.3% to 29.4%)	1.0000
NICU	14	42.8 %		

OBPI = obstetric brachial plexus injury; NICU = neonatal intensive care unit; CI = confidence interval.

**Table 3.** Comparison of PTSD rates in mothers after obstetric brachial plexus injury to mothers of mothers of children in a full-term nursery.

	**N**	**PTSD Event Rate**	**Difference (95% CI)**	***P*-Value**
OBPI	24	41.7%	36.4% (14.3% to 58.5%)	0.004
Full-term Nursery	19	5.3%		

OBPI = obstetric brachial plexus injury; CI = confidence interval.

**Table 4.** Comparison of depression rates in mothers after obstetric brachial plexus injury to mothers of children in a neonatal intensive care unit and mothers of children in a full-term nursery.

	**N**	**Depression Event Rate**	**Difference (95% CI)**	***P*-Value**
OBPI	14	14.3 %	−1.1% (−29.1% to 26.6%)	1.0000
NICU	13	15.4 %		

OBPI = obstetric brachial plexus injury; NICU = neonatal intensive care unit; CI = confidence interval.

**Table 5.** Comparison of depression rates in mothers after obstetric brachial plexus injury to mothers of children in a full-term nursery.

	**N**	**Depression Event Rate**	**Difference (95% CI)**	***P*-Value**
OBPI	14	14.3%	14.3% (−7.2% to 35.2%)	0.1723
Full-term Nursery	19	0.0%		

OBPI = obstetric brachial plexus injury, CI = confidence interval.

## Discussion

A shoulder dystocia delivery with subsequent OBPI can be a difficult and stressful experience for parents and family members. A multidisciplinary approach is required to help treat all facets of the disease process, which evidenced by this study needs to include providing mental health support for families of newborn children with OBPI.

Newborns and infants with OBPI often require intensive physical/occupational therapy, multiple physician visits and may require surgical intervention. These experiences coupled with the necessary work/life adjustments may create additional negative emotions on top of the typical effects of having a newborn, including decreased sleep and altered child care / work schedules. McLean et al. reported 19% of women have poor adjustments to having a newborn with an OBPI [13]. Alyanak and colleagues reported statistically higher rates of depression, attention deficit, anxiety and decreased quality of life in mothers of OBPI newborns [1]. Additionally, Yau and colleagues utilised utilities scores to determine that parents of children with permanent OBPI had worse quality of life when compared to the general population [15].

The purpose of this study was to determine the incidence of depression and PTSD in mothers of a newborn with an OBPI. Fifteen percent screened in for depression symptoms and 42% for PTSD symptoms, which were significantly higher than mothers with a healthy newborn. The general prevalence of PTSD in the United States is 3.5%, and mainly diagnosed in populations with increased risk of trauma such as veterans, police officers and survivors of rape or captivity [16]. Mothers have synonymously associated shoulder dystocia deliveries to assault with devastating consequences [9]. Birth trauma should be recognised as a precipitating life event, which can lead to PTSD, resulting in implications for the individual's functioning in ‘social, interpersonal, developmental, educational, physical health, and occupational domains’ and can be associated with poor family relationships [16]. Only in more recent years have clinicians and researchers begun to acknowledge childbirth as a traumatic event, which can lead to the development of mental disorders like PTSD [17]. Additionally, the mental health of the parent, particularly the mother, can impact their child's physical and mental health resulting in negative outcomes. [3] Additionally, the chronic stress of having to cope with the demands of a child with a birth defect may affect the functioning of the entire family and, ultimately, exacerbate the child's own issues.

There are several limitations of this study. First, this is a single academic institutional study, and results have not been obtained yet to determine reproducibility in other hospital settings. All of the mothers in the current study who screened in for depression and PTSD symptoms were provided mental health resources led by our clinical social workers. However, we did not perform follow-up surveys, and therefore we are unable to comment on the benefits, or lack thereof, of treatment. Furthermore, although the screening criteria were met and mental health resources were administered, there was no confirmation of diagnoses by a clinician. Thus, only risk of depression and PTSD and relative symptoms were able to be assessed. Lastly, due to the small sample size, we were not able to correlate the severity of OBPI with mental health symptoms.

Healthcare providers who treat brachial plexus injuries need to be aware of the social and mental health implications of having a child with an OBPI. OBPI clinics should be staffed with clinical social workers who can screen and help treat depression and PTSD. This multifaceted approach is vital in holistically treating the entire disease process, which includes the mental health component.

### Key Points

- 15% of mothers of a newborn with an OBPI screened in for depression and 42% for PTSD, which were significantly higher than mothers with a healthy newborn.
- Healthcare providers who treat brachial plexus injuries need to be aware of the social and mental health implications of having a child with an OBPI.
- OBPI clinics should be staffed with clinical social workers who can screen and help treat depression and PTSD.
- This multifaceted approach is vital in holistically treating the entire disease process, which includes the mental health component.

## References

[1] Alyanak B, Kılınçaslan A, Kutlu L, Bozkurt H, Aydın A (2013). Psychological adjustment, maternal distress, and family functioning in children with obstetrical brachial plexus palsy. J Hand Surg Am.

[2] Annika J, Paul U, Anna-Lena L (2019). Obstetric brachial plexus palsy – A prospective, population-based study of incidence, recovery and long-term residual impairment at 10 to 12 years of age. Eur J Paediatr Neurol.

[3] Cook N, Ayers S, Horsch A (2018). Maternal posttraumatic stress disorder during the perinatal period and child outcomes: A systematic review. J Affect Disord..

[4] Van Sieleghem S, Danckaerts M, Rieken R, Okkerse JME, de Jonge E, Bramer WM (2022). Childbirth related PTSD and its association with infant outcome: A systematic review. Early Hum Dev.

[5] Heyne CS, Kazmierczak M, Souday R, Horesh D, Lambregtsevan den Berg M, Weigl T (2022). Prevalence and risk factors of birth-related posttraumatic stress among parents: A comparative systematic review and meta-analysis. Clin Psychol Rev.

[6] Beck CT, Watson S, Gable RK (2018). Traumatic childbirth and its aftermath: Is there anything positive?. J Perinat Educ..

[7] Ertan D, Hingray C, Burlacu E, Sterlé A, El-Hage W (2021). Post-traumatic stress disorder following childbirth. BMC Psychiatry..

[8] Beck CT (2009). The Arm: There is no escaping the reality for mother of children with obstetric brachial plexus injuries. Nurs Res..

[9] Beck CT (2013). The obstetric nightmare of shoulder dystocia: a tale from two perspectives. MCN Am J Matern Child Nurs..

[10] Bellew M, Kay SP (2003). Early parental experiences of obstetric brachial plexus palsy. J Hand Surg Br..

[11] Firat T, Oskay D, Akel BS, Oksuz C (2012). Impact of obstetrical brachial plexus injury on parents. Pediatr Int..

[12] Louden E, Allgier A, Overton M, Welge J, Mehlman CT (2015). The impact of pediatric brachial plexus injury on families. J Hand Surg Am..

[13] McLean LA, Harvey DHP, Pallant JF, Bartlett JR, Mutimer KLA (2004). Adjustment of mothers of children with obstetrical brachial plexus injuries: Testing a risk and resistance model. Rehabil Psychol..

[14] Beck CT (2016). Posttraumatic Stress Disorder After Birth: A Metaphor Analysis. MCN: Am J Maternal/Child Nursing.

[15] Yau CWH, Pizzo E, Prajapati C, Draycott T, Lenguerrand E (2018). Obstetric brachial plexus injuries (OBPIs): Health-related quality of life in affected adults and parents. Health Qual Life Outcomes..

[16] American Psychiatric Association (2013). Diagnostic and statistical manual of mental disorders.

[17] Ayers S, Sawyer A (2019). The impact of birth on women's health and wellbeing. Pathways and barriers to parenthood.

